# Navon’s classical paradigm concerning local and global processing relates systematically to visual object classification performance

**DOI:** 10.1038/s41598-017-18664-5

**Published:** 2018-01-10

**Authors:** Christian Gerlach, Nicolas Poirel

**Affiliations:** 1 Department of Psychology, University of Southern Denmark, Odense, Denmark; 2 LaPsyDÉ, UMR 8240, CNRS, Université Paris Descartes, Université Caen Normandie, Paris, France; 3 Institut Universitaire de France (IUF), Paris, France

## Abstract

Forty years ago David Navon tried to tackle a central problem in psychology concerning the time course of perceptual processing: Do we first see the details (local level) followed by the overall outlay (global level) or is it rather the other way around? He did this by developing a now classical paradigm involving the presentation of compound stimuli; large letters composed of smaller letters. Despite the usefulness of this paradigm it remains uncertain whether effects found with compound stimuli relate directly to visual *object* recognition. It does so because compound stimuli are not actual objects but rather formations of elements and because the elements that form the global shape of compound stimuli are not features of the global shape but rather objects in their own right. To examine the relationship between performance on Navon’s paradigm and visual object processing we derived two indexes from Navon’s paradigm that reflect different aspects of the relationship between global and local processing. We find that individual differences on these indexes can explain a considerable amount of variance in two standard object classification paradigms; object decision and superordinate categorization, suggesting that Navon’s paradigm does relate to visual object processing.

## Introduction

Forty years ago David Navon tried to tackle a central problem concerning the course of perceptual processing^1^: “*Do we perceive a visual scene feature-by-feature? Or is the process instantaneous and simultaneous as some Gestalt psychologists believed? Or is it somewhere in between?*” (p. 353). To examine this, Navon developed a now classical paradigm, which involved the presentation of compound stimuli; a large letter (global level) composed of smaller letters (local level) in which the global and the local letters could be the same (consistent) or different (inconsistent). He^1^ found two effects which he argued supported “*..the notion that global processing is a necessary stage of perception prior to more fine-grained analysis*” (p. 371): (i) responses to the global level were faster than responses to the local level, and (ii) when the levels were inconsistent, information at the global level interfered with (slowed down) responses to the local level, but not the other way around.

Since its introduction, Navon’s paradigm has been used in numerous studies, and while different effects may be obtained with this paradigm depending on exposure duration, masking, letter spacing, attentional demands etc.^2–4^, three effects are usually found: (i) a *global precedence effect* with faster judgement of global compared with local shape identity, (ii) an *interference effect* with slower responses to inconsistent than consistent stimuli, and (iii) an *inter-level interference effect* with greater interference effects on local compared with global identity trials.

Navon’s finding of a global shape advantage seems to fit with findings from other paradigms suggesting a coarse-to-fine temporal dynamic in visual object processing^5–11^, and patients with visual recognition deficits following brain damage have also been found to perform abnormally in this paradigm^12,13^. This suggests that Navon’s paradigm may tap some of the same operations that underlie visual *object* processing. This is not a trivial aspect considering the fact that compound stimuli, as also pointed out by Navon^3^, differ from common objects in at least two important respects. First, compound stimuli are not actual objects but rather formations of elements. Secondly, compound stimuli were developed to investigate global and local information processes independently. Consequently, the elements that constitute the global shape are not features of the global shape but objects in their own right (*i.e*., the information present at the global level cannot be predicted from the information present at the local level, or vice-versa).

Even though the evidence considered above seems to suggest that performance in Navon’s paradigm may relate to visual object recognition, this evidence is rather indirect. Hence, while there have been a few group-based studies examining how attention to the global and local level of compound stimuli may bias subsequent objet processing^14,15^, we are unaware of any published studies which have demonstrated that global precedence and interference effects in Navon’s paradigm relate directly to neurological intact individuals’ ability to recognize common objects. This is surprising considering that Navon’s paradigm is so classical that it can be found in almost any cognitive psychology textbook chapter on visual object recognition as a testimony of global shape primacy (see e.g., Kimchi^16^). The main objective of the present study is thus to examine if performance differences among neurologically intact individuals on Navon’s paradigm relate systematically to differences in their ability to recognize common objects such as dogs, cars etc.

As mentioned above, Navon^1^ argued that the global shape advantage was reflected by two aspects of performance with compound stimuli: (i) that responses were faster to the global than the local level, *and* (ii) that the global level interfered with responses to the local level (global-to-local interference). Even though both effects reflect the primacy of global shape characteristics in perceptual processing, there is reason to believe that these effects do not reflect the same underlying operation but may actually reflect two distinct mechanisms^17–19^. In particular, it has been suggested that “sensory mechanisms” (i.e., the magnocellular visual pathway) seems responsible for the faster global processing, whereas “cognitive mechanisms” (i.e., identification processes) seems responsible for the global interference effect^19^.

Evidence supporting a dissociation also comes from studies of patients with brain injury suggesting that these effects can doubly dissociate^20^. We, for example, have described a patient with integrative agnosia who responded faster to the global than the local level of compound stimuli but did not show any interference effects^13^. A similar pattern was reported by Humphreys, *et al*.^21^ in the integrative agnosic HJA. This contrasts with the performance of a developmental prosopagnosic who did show a global-to-local interference effect within the range of normal individuals even though she responded faster to the local than the global level of compound stimuli; a local precedence effect^22^. Similar findings have been reported for other developmental prosopagnosics^23^.

The double dissociation described above suggests that Navon’s paradigm may tap different aspects of global bias: (i) the extent to which global shape characteristics are processed prior to local shape characteristics; an effect we will simply refer to as the *global precedence effect*, and (ii) the extent to which global shape dominates when information from the global and the local levels are combined; an effect we refer to as the *global-to-local interference effect*. Hence, the second objective of the present study is to examine whether and how these effects vary with visual object recognition performance.

The possibility that both the global precedence effect and the global-to-local interference effect are likely to be systematically related to visual object processing performance, even though the effects may reflect different mechanisms, is anticipated in the theoretical framework of the PACE model of visual object processing^22,24^. In this model, visual object processing is based on two main operations: *shape configuration* and *selection*. Shape configuration refers to the binding of visual elements into elaborate shape descriptions in which relationships between the parts are specified, whereas selection refers to the matching of visual impressions to representations stored in visual long-term memory (VLTM). The matching process is thought of as a race among VLTM representations that compete for selection, and the VLTM representation that matches the configured representation the best according to a given criterion will win the competition; hence be selected. The race is initiated by matching the outline (gestalt) of the stimulus to VLTM representations. This first-pass access to VLTM yields initial hypotheses regarding the likely identity of the stimulus. These hypotheses are then used in a top-down manner to augment the buildup of a more detailed description of the visual impression of the stimulus (i.e. shape configuration), which again serves as input for a more specific match with VLTM representations^25^. The greater the demand placed on perceptual differentiation, the more loops comprising VLTM access and shape configuration are required to reach a successful match between the visual input and VLTM representations (i.e. recognition). From this description it is clear that (fast) derivation of global shape information is rather important in the recognition process because it: (i) facilitates the matching process proper by narrowing down the scope of likely VLTM candidates, and (ii) provides the initial frame in which local details can later be embedded. On this account, the global precedence effect is likely to tap the role that global shape serves in the initial phase of visual object processing; being the vehicle for a quick first-pass access to VLTM representations. In comparison, the global-to-local interference effect is more likely to tap the role that global shape serves when a detailed description of the stimulus is to be formed (shape configuration), providing the frame in which details can be embedded. The latter hypothesis is supported by the behavioral pattern exhibited by the patients with integrative agnosia described above^13,21,26^. They were better at recognizing silhouettes – where the global shape is more salient than the local details (features) – than line drawings, and they could also process details. What they could not do efficiently were to integrate information derived from different spatial scales. Hence, their relatively good global shape processing ability was likely reflected in their normal global precedence effect in Navon’s paradigm. Their impaired ability to integrate information derived from different spatial scales, however, was likely reflected in their missing global-to-local interference effect.

In sum, and based on the PACE model, we conjecture that: (i) the more efficiently an individual utilizes global shape information in the first-pass access to VLTM representations, the larger will this individual’s global precedence effect be in Navon’s paradigm, and (ii) the more efficiently information from different spatial levels are bound by an individual during shape configuration, the larger will this individual’s global-to-local interference effect be in Navon’s paradigm. Accordingly, we expect that individual differences in global precedence and global-to-local interference effects will vary systematically with object classification performance. The magnitude of these relationships, however, will vary as a function of how much a given task tax shape configuration and selection; a point we will address below.

**Figure 1:**
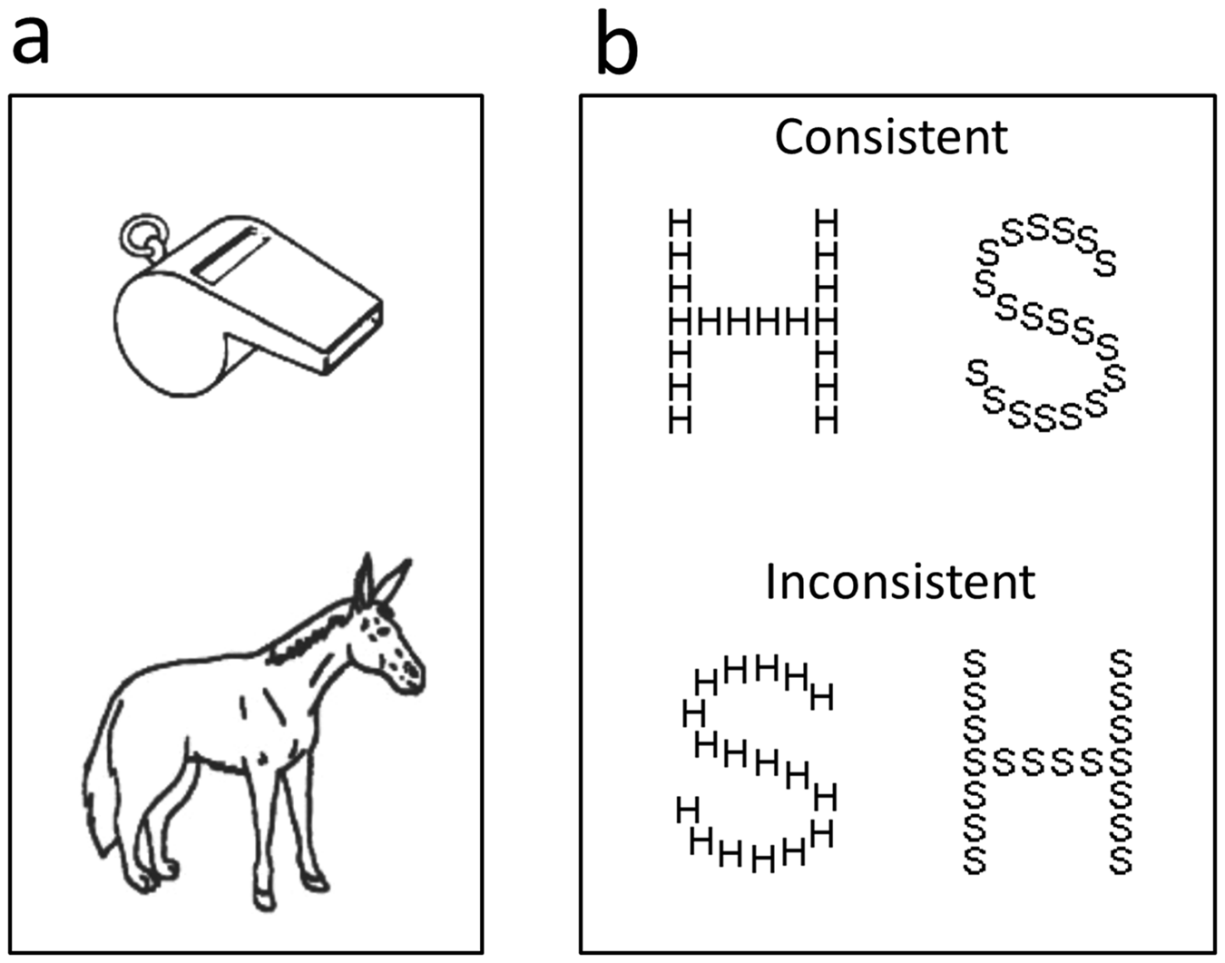
Stimuli. (**a**) Examples of stimuli used in the object decision task (top = real object, bottom = nonobject composed of half a wolf and half mule). Reproduced with kind permission from Lloyd-Jones^36^. (**b**) Stimuli used in Navon’s paradigm.

To examine whether performance on Navon’s paradigm varies systematically with object recognition of common objects we used two different tasks; superordinate categorization and difficult object decision. In the superordinate categorization task – henceforth simply termed *categorization task* – the participants had to decide whether stimuli depicted natural objects or artefacts. In the difficult object decision task, the participants were required to decide whether pictures represented real objects or nonobjects. We term the object decision task “difficult” because the nonobjects were chimeras composed of real objects (see Fig. 1a). This makes the discrimination between real objects and nonobjects harder than if the nonobjects are completely novel^27^.

While both object decision and categorization require object classification, they differ in the amount of structural processing needed for making the classification called for. In the categorization task it may be sufficient to recognize just the global shape (outline) of an object, or a part of it, in order to decide whether it is a natural object or an artefact. As an example, you need not decide whether the target item – for example a cat – is a cow, cat, dog or a horse to categorize it as a natural object. On the contrary, the more similar the stimulus is to other members of the category NATURAL objects, and the less similar it is to members of other categories which are not natural, the higher will be the probability that it belongs to that particular category compared with other categories. The same coarse processing strategy will not suffice in the difficult object decision task because the nonobjects may have similar global shapes as real objects and indeed will contain parts from real objects. Hence, in contrast to categorization, difficult object decision requires that features be sampled from and integrated across the whole stimulus and that a complete match with a VLTM representation is found^13^. Evidence supporting this difference in the demand of perceptual differentiation required by the two tasks can be found in several studies. Gerlach *et al*.^28^, for example, found that difficult object decision was associated with greater activation of posterior and ventral brain regions than categorization; areas associated with structural processing. Likewise, superordinate categorizations can be performed a lot faster than difficult object decisions^29–31^.

Based on the considerations presented above we hypothesize the following outcomes: (i) Individual differences in global precedence effects will vary systematically with performance on both the object decision and the categorization task because global shape processing is involved in both, and (ii) that individual differences in global-to-local interference effects will vary systematically with performance on the objet decision task but not necessarily with performance on the categorization task because categorization requires less perceptual differentiation than difficult object decision and therefore does not tax shape configuration to any great extent. Consequently, we expect: (i) a negative correlation between global precedence effects and reaction time (RT) in both the object decision and the categorization tasks – the larger the global precedence effect the faster the RTs – and (ii), a negative correlation between global-to-local interference effects and RT in the object decision task, with larger interference effects being associated with faster RTs.

## Results

### Object decision and categorization

The mean correct RT in the object decision task was 835 ms (95% CI [796, 873]) and 656 ms in the categorization task (95% CI [629, 682]). This difference between tasks was significant (*M*_*dif*_ *= *179 ms, 95% CI [149, 209], *p* < 0.0001, *d*_z_ = 1.1).

The mean % correct responses in the object decision task was 95.5 (95% CI [94.9, 96.1]) and 98.3 in the categorization task (95% CI [97.9, 98.6]). This difference between tasks was also significant (*M*_*dif*_ *= *2.8%, 95% CI [2.1, 3.4], *p* < 0.0001, *d*_z_ = 0.8).

### Navon’s paradigm

The RTs for all participants were subjected to a two-way ANOVA with Level (Global vs. Local identity judgements) and Consistency (Consistent vs. Inconsistent stimuli) as factors. This analysis revealed a significant main effect of Level (*F*(1,115) = 410, partial η^2^ = 0.78, *p* < 0.0001), with faster global compared with local identity judgements, a main effect of Consistency (*F*(1,115) = 439, partial η^2^ = 0.79, *p* < 0.0001), with faster responses to consistent than inconsistent stimuli, and an interaction between Level and Consistency (*F*(1,115) = 309, partial η^2^ = 0.73, *p* < 0.0001), with the difference in responses to consistent and inconsistent stimuli being larger on local than on global trials. Post-hoc analyses revealed significant differences for: Global Inconsistent vs Global Consistent (*t*_115_ = 6.8, *p* < 0.0001), Local Inconsistent vs. Local Consistent (*t*_115_ = 23.6, *p* < 0.0001), Local Consistent vs. Global Consistent (*t*_115_ = 13.2, *p* < 0.0001), and Local Inconsistent vs. Global Inconsistent (*t*_115_ = 24.2, *p* < 0.0001). Hence, the interaction reflected that the difference in responses to consistent and inconsistent stimuli was larger on local than on global trials (i.e. [Global Inconsistent RTs - Global Consistent RTs] vs. [Local Inconsistent RTs minus Local consistent RTs], 14 ms vs. 68 ms respectively, *t*_115_ = 17.6, *p* < 0.0001). See Table 1 for details concerning mean RT and accuracy for each of the four conditions.

**Table 1:** Mean correct RT (ms) and % correct responses for each of the four conditions in Navon’s paradigm. SD’s are given in brackets.

	RT	% Correct responses
Global consistent trials	445 (71)	94.5 (3.9)
Global inconsistent trials	459 (76)	92.7 (5.7)
Local consistent trials	500 (81)	96.2 (4.8)
Local inconsistent trials	568 (90)	90.5 (7.4)

### Relationship between performance on Navon’s paradigm and visual object processing

In order to examine whether individual differences in global processing revealed by Navon’s paradigm are systematically related to individual differences in visual object classification performance, we first derive two indexes from Navon’s paradigm which reflect different aspects of global shape processing. The first index, which we term the *Global-Local Precedence index*, is based on the difference in RT to global and local identity judgements on consistent trials. Positive values on this index reflect faster processing of global compared with local shape information. The second index, which we term the *Global-to-Local Interference index*, is based on the difference in RT to local identity judgements on inconsistent and consistent trials. Positive values on this index reflect that global shape information dominates over (interferes with) local shape information when information from both levels must be combined. Both indexes are computed for each participant as a standardized mean difference (Cohen’s *d*), that is, as the difference between the two means of interest divided by their pooled standard deviations. As we have shown previously^22^, using such standardized measures yield more reliable estimates than measures based on only the absolute differences between means (which disregard the variance). This aspect is rather important in the present context because poor reliabilities of two measures are likely to distort the correlation that may be obtained between them^32^. Hence, prior to conducting the correlation analyses between our two measures of visual object classification – object decision and categorization – and our two measures of global shape processing, we first computed the Spearman-Brown-corrected split-half reliability of the four measures by comparing values on odd and even trials for each measure. The reliability of the measures were 0.81 for the Global-Local Precedence index, 0.66 for the Global-to-Local interference index, 0.97 for the object decision task, and 0.98 for the categorization task; figures which we deem satisfactory^33^.

**Figure 2:**
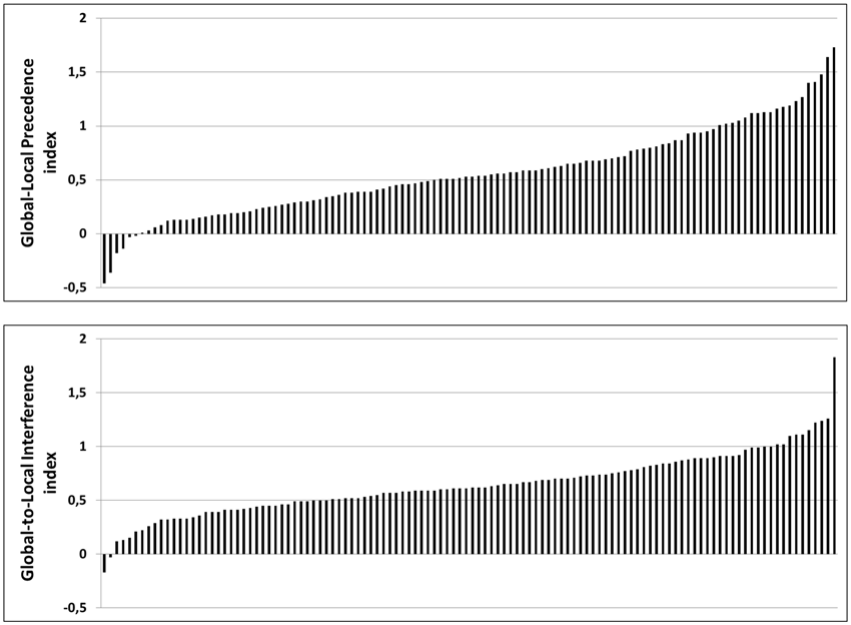
Individual scores on the two indexes of global/local processing. Upper panel: Scores for each participant on the Global-Local Precedence index. Lower panel: Scores for each participant on the Global-to-Local Interference index. Note that scores in each panel are ordered according to magnitude and not by individual.

The mean score on the Global-Local Precedence index was 0.56 (*SD* = 0.41, Range: −0.46–1.73), reflecting that responses were generally faster for global than for local identity judgements. As can be seen from Fig. 2 (upper panel), this was true for the majority of the participants (95%). The mean score on the Global-to-Local Interference index was 0.64 (*SD* = 0.29, Range: −0.17–1.83), reflecting that global shape information interfered with report of local shape identity. Again, this was true for the majority of the participants (98%) (see Fig. 2, lower panel).

To examine the relationship between the Global-Local Precedence index and the Global-to-Local Interference index, we used the Pearson product-moment correlation coefficient and estimated the correlation coefficient CIs by means of bias corrected and accelerated bootstrap analyses with 1000 samples. This revealed no reliable correlation between the measures (*r = *0.04, 95% CI = [0.20, −0.12]). This provides further evidence for the notion, discussed in the introduction, that global precedence effects and global-to-local interference effects reflect different mechanisms.

To examine potential relationships between the Navon indexes and visual object classification performance, we used the Pearson product-moment correlation coefficient and estimated the correlation coefficient CIs by means of bias corrected and accelerated bootstrap analyses with 1000 samples.

**Figure 3:**
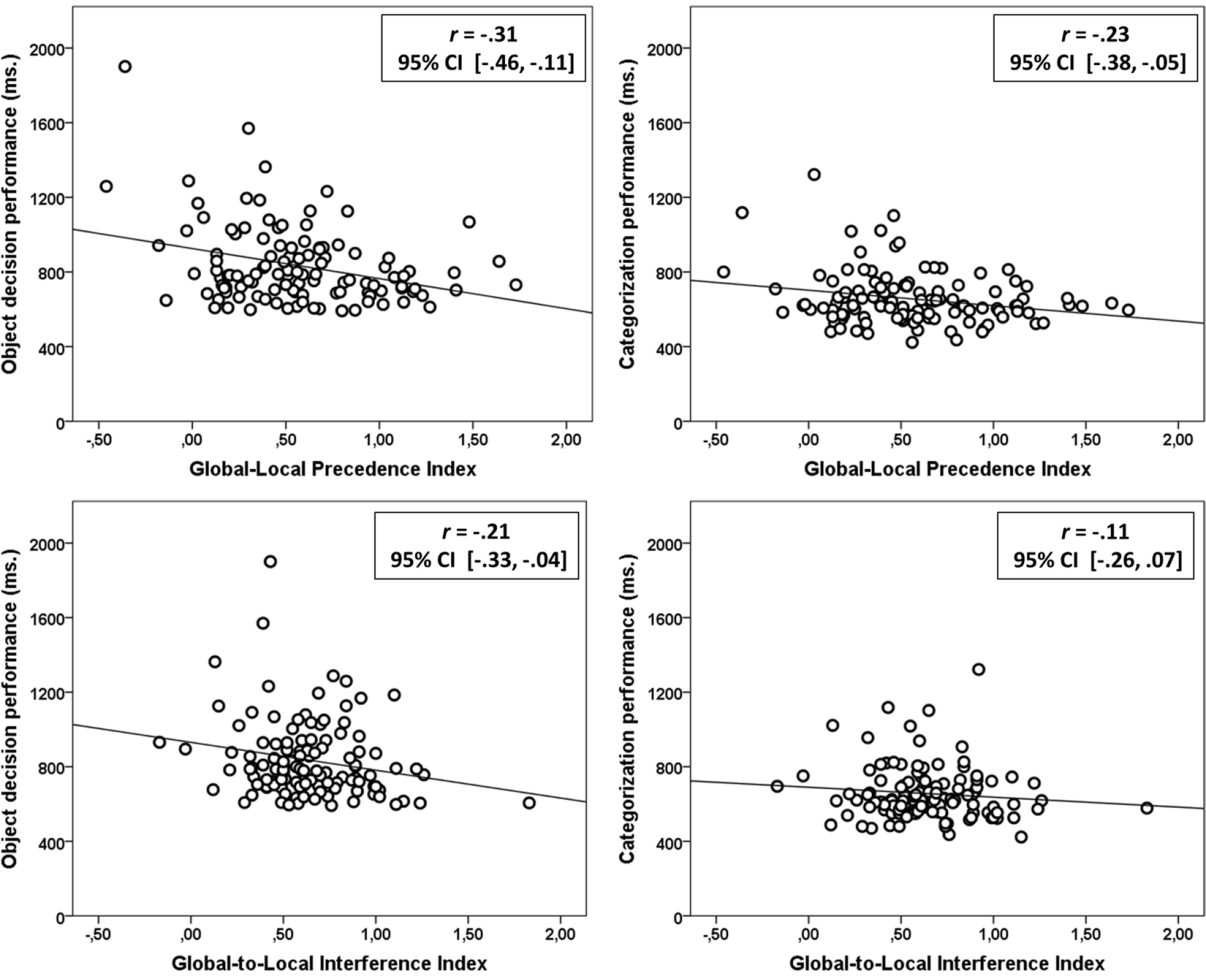
Correlations between the global/local indexes and task performance. Scatterplots showing the relationship between: the Global-Local Precedence index and object decision performance (upper panel left), the Global-Local Precedence index and categorization performance (upper panel right), the Global-to-Local Interference index and object decision performance (lower panel left), and the Global-to-Local Interference index and categorization performance (lower panel right). Also shown are the regression lines, the Pearson correlation coefficients (*r*) and their associated 95% CI’s.

The Global-Local Precedence index correlated negatively with RT in both the object decision (*r = *−0.31, 95% CI = [−0.46, −0.11]) and the categorization task (*r = *−0.23, 95% CI = [−0.38, −0.05]) (See Fig. 3, upper panels): The greater the global bias, the more efficient the object classification performance. While the correlation was numerically higher for object decision than for categorization this difference was not significant (*t*_*Difference*_ = −1.05, *p* = 0.15). Using the Global-Local Precedence index as a predictor variable for object decision and categorization performance yielded no more than 5% of cases having standardized residuals larger than 1.96, suggesting that both models provided reasonable fits the to the majority of the data. Also, for no case was Cook’s distance larger than 0.63, which also suggests that no single case had an undue influence on the models as a whole.

The Global-to-Local Interference index correlated negatively with object decision RT (*r = *−0.21, 95% CI = [−0.33, −0.04]) (See Fig. 3, lower panel left): The greater the global-to-local interference, the more efficient the object decision performance. In comparison, the correlation between the Global-to-Local Interference index and categorization RT was not reliable (*r = *−0.11, 95% CI = [−0.26, 0.07]) (See Fig. 3, lower panel right). Using the Global-to-Local Interference index as a predictor variable for object decision performance yielded no more than 5% of cases having standardized residuals larger than 1.96, suggesting that the model provided a reasonable fit the to the majority of the data. Also, for no case was Cook’s distance larger than 0.17, which also suggests that no single case had a critically high influence on the model as a whole.

To examine the relative predictive value of the Global-Local Precedence index and the Global-to-Local Interference index on object decision performance, we conducted a hierarchal multiple regression analysis with two steps. Step one included only the Global-Local Precedence index as a predictor of object decision performance, whereas step two also included the Global-to-Local Interference index as a predictor. As can be seen from Table 2, both the Global-Local Precedence index and the Global-to-Local Interference index were reliable and independent predictors of object decision RT, and hence addition of the Global-to-Local Interference index increased the fit of the regression model from *R* ^2^ = 0.1 to *R* ^2^ = 0.14.

**Table 2:** Linear model of predictors of object decision performance (RT). 95% confidence intervals and standard errors are estimated by means of bias corrected and accelerated bootstrap analyses with 1000 samples.

	*b*	95% CI	*SE b*	β
Step 1				
Constant	925	847, 1008	46	
Global-Local Precedence index	−161	−304, 143	60	−0.31
Step 2				
Constant	1014	901, 1132	66	
Global-Local Precedence index	−157	−294, −43	59	−0.31
Global-to-Local Interference index	−141	−259, −11	59	−0.19

Note. *R* ^2^ = 0.1 for Step 1; *∆R* ^2^ = 0.14 for Step 2 (*p*’s < 0.05).

## Discussion

The main objective of the present study was to investigate if performance on Navon’s paradigm relates systematically to the ability to recognize commons objects in two standard object classification paradigms (object decision and superordinate categorization). The results confirmed that the tasks worked as intended. As expected, the Navon paradigm gave rise to: (i) a global precedence effect, with faster judgements of the identity of global compared with local elements, (ii) a consistency effect, with faster responses to consistent than inconsistent stimuli, and (iii) an inter-level interference effect with greater effects of consistency on local compared with global identity trials. Likewise, RTs were significantly higher and accuracy significantly lower in the object decision task compared with the categorization task. This supports the assumption that classifying an object at a superordinate level requires less object individuation (perceptual differentiation) than deciding whether it represents a real object or a chimeric nonobject^25,28^.

Of more interest is the finding that individual differences on the Global-Local Precedence index do vary systematically with both object decision and superordinate categorization performance. This suggests that the global precedence effect reflects something that is common for visual object recognition tasks in general. As predicted, for both object decision and categorization, faster RTs were associated with larger global precedence effects. Even though the relationship was stronger for object decision than for categorization, this difference between tasks was not significant. Nevertheless, we will offer one speculation for this difference, and it has to do with task requirements.

As argued in the introduction, categorization at a superordinate level can probably be based on the global shape (outline) of an object, or a just part of it, whereas such a coarse processing strategy will not suffice in the difficult object decision task because the nonobjects may have similar global shapes as real objects and indeed will contain parts from real objects. Hence, it may be that difficult objects decision is based on global shape information in addition to local shape information (features) whereas categorization may occasionally be based on recognition of features alone. The net result would be that global shape would generally play an important part in both categorization and object decision, but a more significant part in object decision, thus explaining why global precedence effects may vary more systematically with object decision than with categorization.

While this explanation amounts to nothing more than a post-hoc speculation regarding a non-significant finding, it is precisely an explanation along these lines that led us to hypothesise that individual differences in global-to-local *interference* effects would vary systematically with object decision performance but not necessarily with categorization performance. The rationale was that if categorization can be based on coarse processing without the need to bind global and local shape information prior to classification, whereas object decision cannot, we should expect individual differences in global-to-local interference effects – which reflect the binding of global and local shape characteristics – to vary systematically with object decision but not with categorization performance. This prediction was borne out. In addition, we find that individual differences in global precedence effects and global-to-local interference effects were significant and independent predictors of object decision performance. This supports the notion that while both effects reflect the primacy of global shape characteristics in perceptual processing, they are likely to tap different operations^19,22^.

The finding that individual differences in global precedence effects can account for performance differences in both object decision and categorization, and that global precedence effects and global-to-local interference effects can account for different aspects of individual variation in object decision performance, can be explained by the PACE model of visual object recognition^24,25^; a model which also served to form some of the predictions examined here. This model assumes the existence of two operations in visual object classification; *shape configuration* and *selection*. Shape configuration refers to the binding of visual elements into elaborate shape descriptions in which relationships between the parts are specified, whereas selection refers to the matching of visual impressions to representations stored in visual long-term memory (VLTM). The matching process is thought of as a race among VLTM representations that compete for selection, and the VLTM representation that matches the configured representation the best according to a given criterion will win the competition; hence be selected. The race is initiated by matching the outline (gestalt) of the stimulus to VLTM representations. This first-pass access to VLTM yields initial hypotheses regarding the likely identity of the stimulus. These hypotheses are then used in a top-down manner to augment the buildup of a more detailed description of the visual impression of the stimulus (i.e. shape configuration), which again serves as input for a more specific match with VLTM representations^25^. The greater the demand placed on perceptual differentiation, the more loops comprising VLTM access → shape configuration may be required to reach a successful match between the visual input and VLTM representations (i.e. classification). On this account, the global precedence effect is likely to reflect the efficiency with which global shape processing supports the first-pass access to VLTM representations; an operation which is important for both objet decision and categorization. In comparison, the global-to-local interference effect, which is a product of the binding of global and local shape information, is more likely to reflect the efficiency with which global shape supports the shape configuration stage by providing the frame in which details can be embedded (a process that has to be inhibited in Navon’s paradigm when informations are in conflict^34^); an operation that is important in classification contexts such as difficult object decision where the demand on perceptual differentiation is high.

In conclusion, Navon’s paradigm^1^, with its use of compound stimuli, has been very influential in shaping our understanding of the course of perceptual processing in both normality and clinical populations. One possible limitation of this paradigm, however, concerns whether effects found with compound stimuli relate to visual *object* processing given that compound stimuli differ from common objects in two important respects: (i) Compound stimuli are not actual objects but rather formations of elements, and (ii) the elements that constitute the global shape in compound stimuli are not features of the global shape but rather objects in their own right^3^. The present results suggest that findings obtained with this paradigm do in fact relate rather directly to visual recognition of common objects in that individual differences in global precedence and global-to-local interference effects can explain a substantial amount of variance in two standard object classification paradigms; difficult object decision and superordinate classification. In addition, the present findings indicate that the global precedence effect and the global-to-local interference effect tap different operations in visual object recognition, and we argue that this difference can be explained in the framework of the PACE model of visual object processing^24,25^.

## Method

### Participants

116 subjects participated (mean age 23, *SD* = 5.4, range 19–53 years, 90 females). The participants were first-year students in the psychology programme at the University of Southern Denmark. The participants were naïve to the specific hypotheses tested, and they participated as part of their course in cognitive psychology. The course, including experimental participation by the students, is approved by the study board at the Department of Psychology, University of Southern Denmark, and the experiments conducted do not require formal ethical approval/registration according to Danish Law and the institutional requirements. Prior to participation the students were informed that data collected in the experiments might be used in an anonymous form in future publications. Participants were free to opt-out if they wished, and participation in the experiments was taken as consent.

In all tasks performed, the participants were encouraged to respond as fast and as accurately as possible. Prior to each task the participants performed a practice version of the upcoming task. Stimuli used in these practice versions were not used in the actual experimental conditions. RTs were recorded by means of the keyboard.

### Procedure and stimuli

#### Object Decision

The participants were presented with 160 pictures (80 real objects and 80 nonobjects), and instructed to press the ‘M-key’, if the picture represented a real object and the ‘N-key’, if it represented a nonobject. The 80 pictures of real objects (40 natural objects and 40 artefacts) were taken from the set by Snodgrass and Vanderwart^35^. The 80 chimeric drawings of nonobjects were selected mainly from the set made by Lloyd-Jones and Humphreys^36^. These nonobjects are line-drawings of closed figures constructed by exchanging parts belonging to objects from the same category (see Fig. 1a). The order of pictures was randomized.

All stimuli were presented centrally on a white background on a computer screen and subtended 3–5° of visual angle. The stimuli were displayed until the participant made a response. The interval between response and presentation of the next object was 1 s.

### Categorization task

The participants were presented with 80 pictures of real objects, the same as used in the object decision task, and instructed to press the M-key if the picture represented an artefact and the N-key if it represented a natural object. All stimuli were presented centrally on a white background and displayed until the participant made a response. The interval between response and presentation of the next object was 1 s.

### Navon’s paradigm

The participants were presented with large letters, either ‘H’ or ‘S’, that could consist of either smaller ‘H’s or ‘S’s (see Fig. 1b).

Each participant was presented with four experimental blocks. In two blocks, the participants were required to report the identity of the global letter (by keyboard press of the relevant letter). In the other two blocks, they were to report the identity of the local letters. The blocks were presented in an ABBA design and began with global identity judgements.

The large letters subtended 3.91° × 5.25° of visual angle, and the small letters 0.47° × 0.67° of visual angle. The fixation cross presented before stimulus onset subtended 0.95° × 0.95° of visual angle. All stimuli were black presented on a white background.

Participants performed a total of 80 trials in each block, 40 consistent (same identity of local and global letters) and 40 inconsistent. The stimuli were shown at four different positions on the screen (top, bottom, left, and right) relative to the fixation cross. An equal number of stimuli within each block (*n* = 20: 10 consistent and 10 inconsistent) were presented at each of the four locations. For each location, the stimuli were presented so that the centre of the global shape was positioned 3.34° of visual angle from the fixation cross. The order of position and consistency (consistent vs. inconsistent stimuli) was randomized.

A trial began with a fixation cross presented in the middle of the screen for 1 s, which the participants were instructed to look at when present. This was followed by stimulus onset which was replaced after 180 ms. by a blank screen which remained until response. Before each block, the participants performed 16 practice trials.

The participants first performed the object decision task followed by the categorization task. Finally, following a short break, they performed the Navon task.

All analyses of RTs for the object decision task were based on responses to real objects only as the nonobjects served no other purpose in the present study than to ensure detailed shape processing of the real objects^27^. Likewise, accuracy is also based on responses to real objects only. Given that exactly the same real objects were presented in the object decision task and the categorization task this makes RTs and accuracy directly comparable across tasks.

Prior to statistical analysis the data on correct trials were trimmed for each participant excluding any RT that fell above or below 2½ *SD*s from the mean of a given participant in each of the three tasks. This resulted in the removal of 3.4% trials on average from the object decision task (*SD* = 1.2, range: 1.2–7.5), 2.8% from the categorization task (*SD* = 1.1, range: 0–6.2), and 2.4% from the Navon task (*SD* = 0.8, range: 0.3–4.6); figures which are well within the recommended limits suggested by Ratcliff^37^. Data for these and the other analyses reported can be found in the supplemental data file [Supplementary_Data.xlsx].

## Electronic supplementary material

Supplementary Dataset
